# ID 135 - Implementação de Tecnologias no Sistema Único de Saúde: o caso da biotina para o tratamento da deficiência de biotinidase

**DOI:** 10.5327/2237-9622.2025.v34s1.135

**Published:** 2025-11-25

**Authors:** Adriane Lopes Medeiros Simone, Gabrielle Ferrante Alves de Moraes, Juliana Soprani, Bianca O Cata-Preta, Bruna Bento dos Santos, Stéfani Sousa Borges, Daniela Oliveira de Melo

**Keywords:** biotina, deficiência de biotinidase, Sistema Único de Saúde, Implementação

## Abstract

**Introdução:**

A biotina é uma vitamina do complexo B, obtida por meio da dieta, que serve como coenzima, essencial em processos como gliconeogênese, síntese de ácidos graxos e catabolismo de aminoácidos. A administração oral em sua forma livre é importante no tratamento da deficiência de biotinidase (DB), um distúrbio metabólico raro resultante de erros no metabolismo da biotina. Para que o acesso a um medicamento se concretize em um sistema de saúde, é necessário que etapas de incorporação e implementação de tecnologias sejam cumpridas. O objetivo deste estudo foi analisar o processo de incorporação e de implementação da biotina no Sistema Único de Saúde (SUS).

**Método:**

Foi realizada análise temporal dos eventos relacionados à incorporação e à implementação da biotina para o tratamento da DB em bases de dados oficiais, entre 2012 e 2019.

**Resultados:**

Frente às necessidades envolvendo doenças raras, a Secretaria de Atenção à Saúde demandou a incorporação de biotina para o tratamento da DB em 2012, resultando na produção do Relatório de Recomendação (RR) n.º 06, com recomendação favorável da Comissão Nacional de Incorporação de Tecnologias (Conitec). A incorporação do medicamento ocorreu pela Portaria n.º 34, de 27 de setembro de 2012, com prazo máximo de 180 dias para efetivar a oferta no SUS. Entretanto, somente em 2016 foi realizado o registro de biotina isolada na Agência Nacional de Vigilância Sanitária (AnvisaA), na forma farmacêutica cápsula de 2,5 mg, pela Aché Laboratórios Farmacêuticos, sob o nome comercial Untral®. Em agosto de 2018, foi pactuado na Comissão Intergestores Tripartite a inclusão da biotina no elenco do Componente Especializado da Assistência Farmacêutica (Ceaf), três meses após a publicação do Protocolo Clínico e Diretrizes Terapêuticas (PCDT) de DB. A primeira aquisição pelo Ministério da Saúde aconteceu entre julho e novembro de 2019, mesmo período em que houve a atualização do Sistema de Gerenciamento da Tabela de Procedimentos, Medicamentos e OPM do SUS (Sigtap). A primeira dispensação de biotina aconteceu somente em 2020, no Paraná e em São Paulo.

**Conclusão:**

O RR apoia a decisão de incorporação e a elaboração do PCDT, além de ser utilizado para subsidiar as etapas de implementação e nortear como o medicamento será dispensado ao paciente. A implementação da biotina ocorreu em agosto de 2018, seis anos após a sua incorporação. Estudos complementares são necessários para melhor compreensão do contexto, barreiras e facilitadores envolvidos na implementação desta tecnologia no SUS. Contudo, os resultados sugerem que há uma discrepância entre a política como intenção (o que consta na legislação) e a política como prática (o que realmente é concretizado) no SUS. Nesse sentido, infere-se a necessidade de maior controle e monitoramento desses processos no âmbito do Poder Executivo. O alinhamento e a celeridade nas etapas de incorporação e implementação de tecnologias no SUS é essencial para promoção do acesso aos medicamentos em tempo oportuno pela população.

